# Supplementary material for: Investigation of the effect and mechanisms of moxa smoke in the treatment of Influenza A Virus (IAV) infection
Source: PLoS One. 2025 Dec 12;20(12):e0337906. doi: 10.1371/journal.pone.0337906 (PMC12700386; doi:10.1371/journal.pone.0337906)
Supplement: S1 File — (PDF) [file pone.0337906.s001.pdf]

Fig 8 Western blot

Fig 8

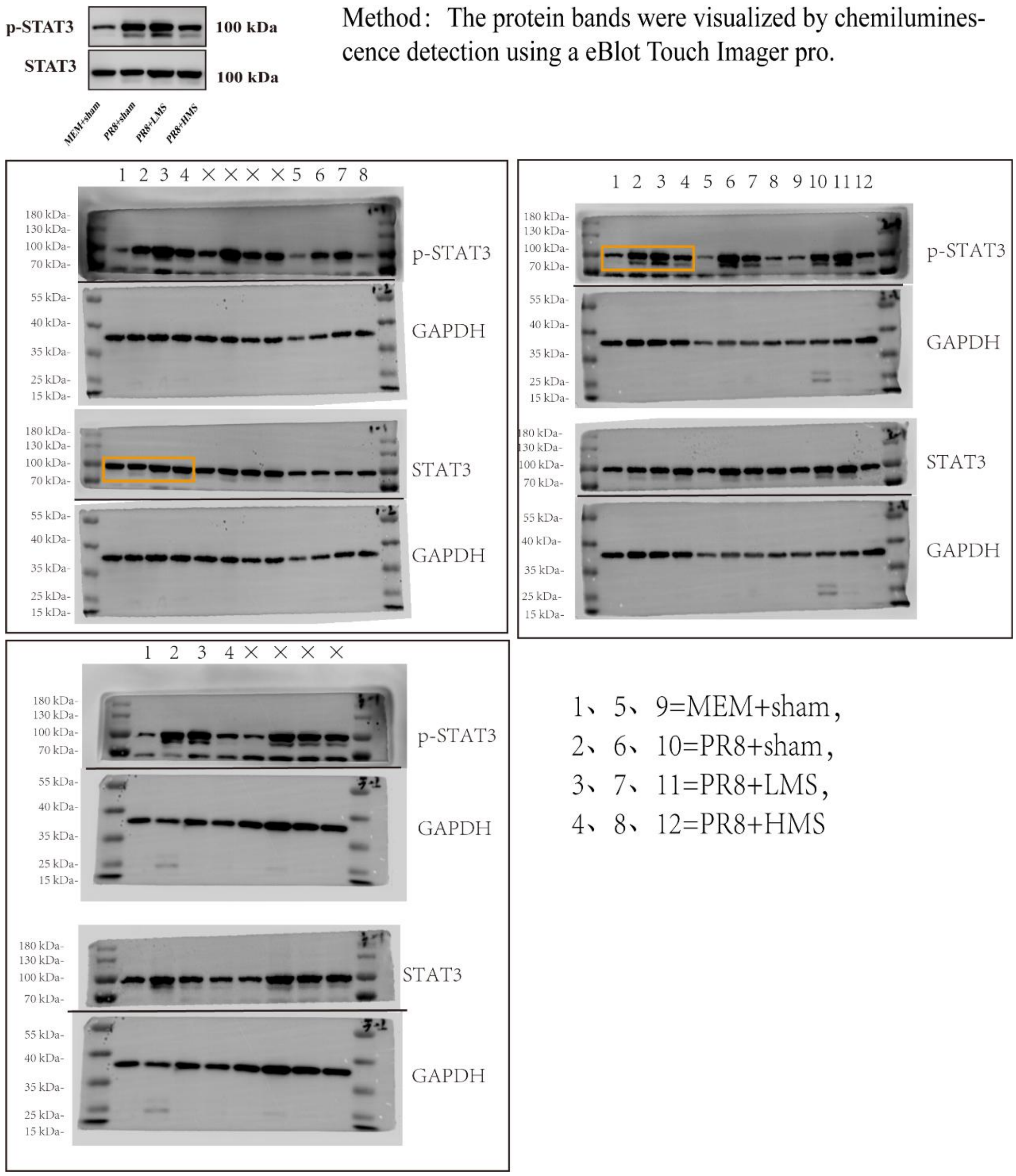

Method: The protein bands were visualized by chemiluminescence detection using a eBlot Touch Imager pro.

1、5、9=MEM+sham,
2、6、10=PR8+sham,
3、7、11=PR8+LMS,
4、8、12=PR8+HMS

Fig 8

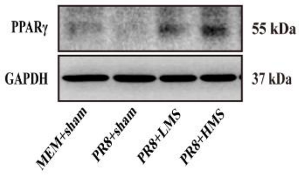

Method: The protein bands were visualized by chemiluminescence detection using a Bio-Rad ChemiDoc Touch imaging system.

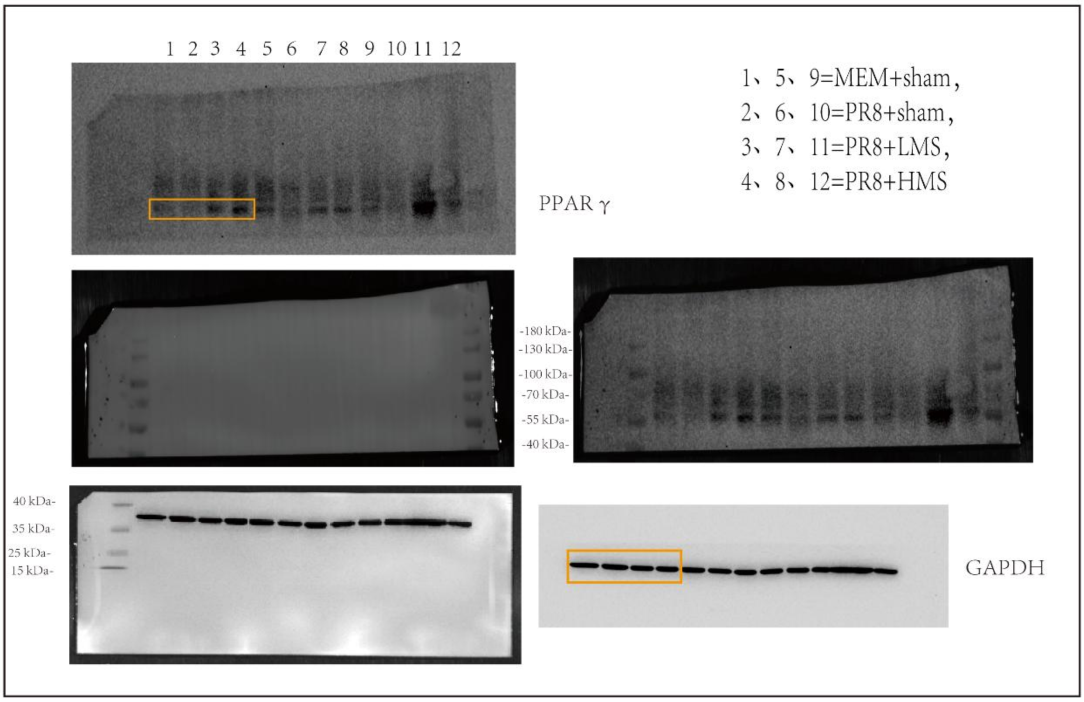

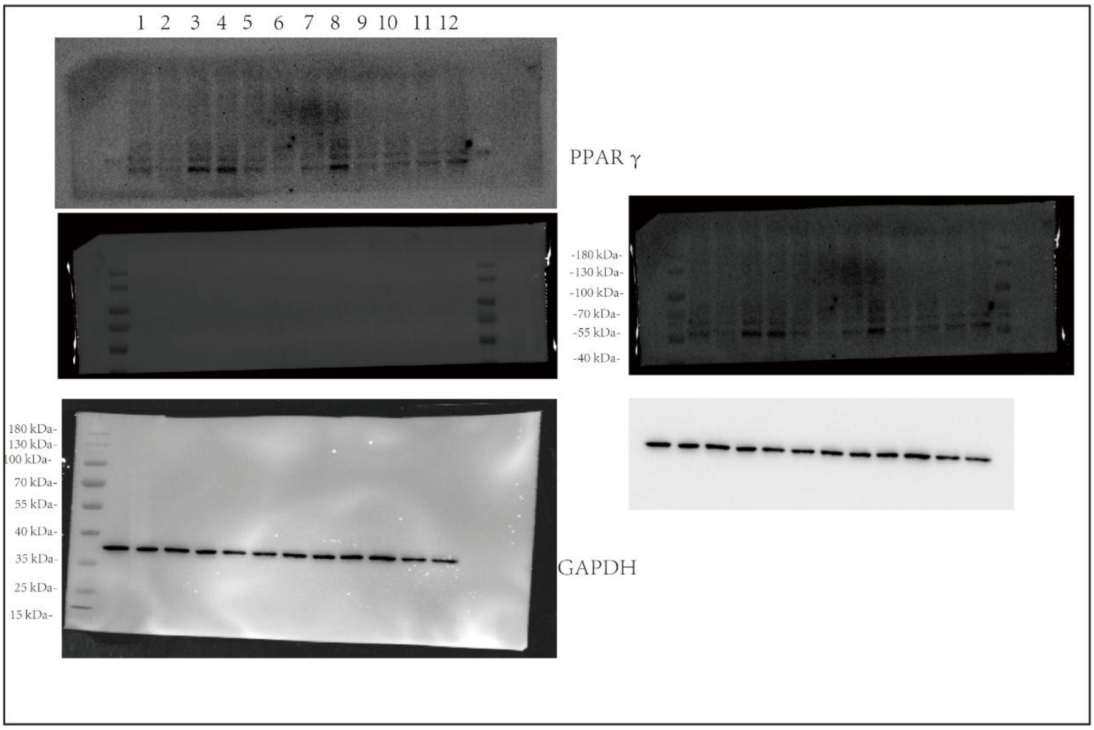

PPARG and the loading control (GAPDH) were detected on separate gels under optimized conditions. The intensity of the bands was measured using Image Lab

software, and the expression levels of the target protein were normalized to the loading control and compared with the control group. All samples originated from the same batch and were loaded in equal amounts.

Chemiluminescent detection and densitometric analysis were initially carried out using a Bio-Rad gel imaging system along with its proprietary Image Lab software. After the Bio-Rad instrument was retired, later images were captured using an EBlot chemiluminescent imager. These EBlot images were then analyzed with Image J software. Crucially, the core quantification method—measuring band intensity, normalizing to the loading control, and determining relative expression compared to the control group—remained the same across both software platforms, ensuring that all data remained comparable.

Fig 9C

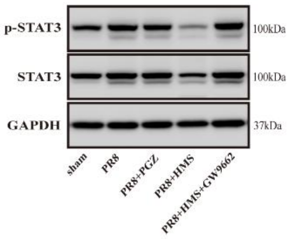

Method: The protein bands were visualized by chemiluminescence detection using a eBlot Touch Imager pro.

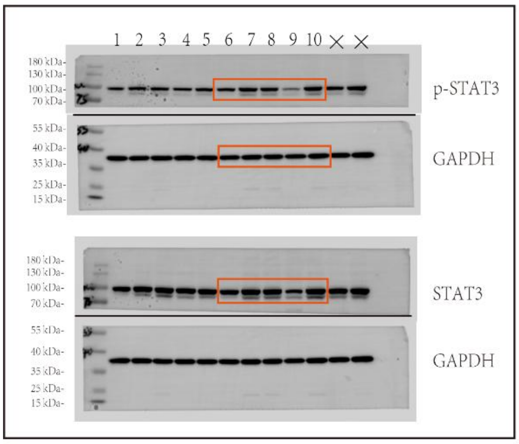

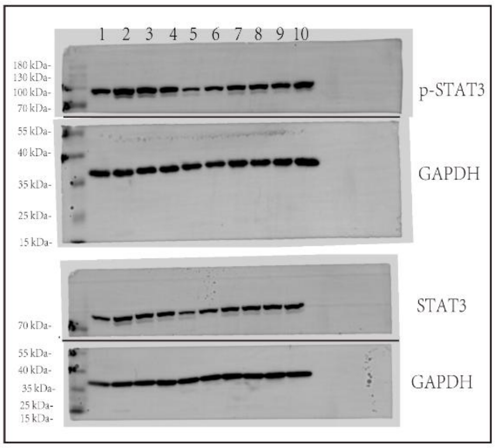

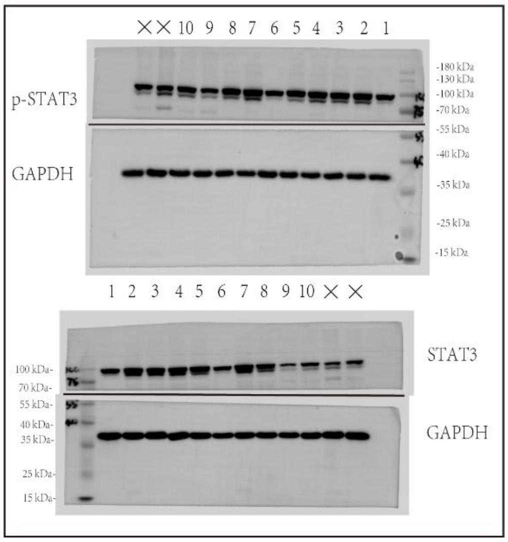

1、6=sham
2、7=PR8
3、8=PR8+PGZ
4、9=PR8+HMS
5、10=PR8+HMS+GW9662
