# Supplementary material for: Investigation of the effect and mechanisms of moxa smoke in the treatment of Influenza A Virus (IAV) infection
Source: PLoS One. 2025 Dec 12;20(12):e0337906. doi: 10.1371/journal.pone.0337906 (PMC12700386; doi:10.1371/journal.pone.0337906)
Supplement: Attachment — Submitted filename: PONE-D-25-21289.docx [file pone.0337906.s003.docx]

Overall Evaluation

This study explores the antiviral and anti-inflammatory effects of moxa smoke (MS) against H1N1 infection using network pharmacology, molecular docking, and in vivo experiments. The integration of traditional Chinese medicine (TCM) with modern pharmacological approaches is commendable, offering novel insights into MS’s potential therapeutic applications. However, while the research demonstrates scientific rigor in certain aspects, it contains critical gaps, particularly regarding clinical safety implications and mechanistic clarity, which must be addressed for publication.

Strengths and Innovations

1. Multidisciplinary Approach to Mechanism DiscoveryThe study employs network pharmacology to systematically identify MS components (52 compounds) and their potential targets (384 proteins), uncovering 16 core targets (e.g., STAT3, PPARγ) via PPI network analysis. This “multi-component, multi-target” framework aligns with TCM’s holistic philosophy and provides a robust basis for understanding MS’s complex actions. The molecular docking validation of stable binding between MS compounds (e.g., Bis-(3,5,5-trimethylhexyl) phthalate) and key targets further strengthens the mechanistic hypothesis.

2. In Vivo Validation of Antiviral and Anti-Inflammatory EffectsUsing a PR8-infected mouse model, the study demonstrates that MS reduces viral load (H1N1NP mRNA), alleviates pulmonary inflammation (e.g., decreased BALF leukocytes, IL-6/TNF-α expression), and modulates PPARγ/STAT3 pathways. These findings provide experimental evidence for MS’s therapeutic potential, particularly its ability to suppress cytokine storms—a critical pathology in severe influenza.

3. Translational Relevance to TCM PracticeBy focusing on MS, a byproduct of moxibustion with historical use in infection prevention, the study bridges traditional practices with modern science. The identification of PPARγ as a key regulator offers a mechanistic explanation for MS’s anti-inflammatory effects, potentially guiding the development of targeted TCM interventions.

Critical Weaknesses and Safety Concerns

1. Inadequate Assessment of Clinical Safety Risks

Occupational Exposure Risks for Acupuncturists:The manuscript fails to address the chronic toxicity of MS, a major concern for 针灸医师 (acupuncturists) and patients. Existing evidence (cited in prior reviews) shows that long-term exposure to moxa smoke is associated with increased incidence of chronic pharyngitis, allergic reactions, and potential pulmonary fibrosis. For example, elevated MMP12 expression in MS-treated mice (Table 3) indicates ECM degradation, a hallmark of emphysema and pulmonary fibrosis. The study’s failure to discuss this finding’s clinical relevance constitutes a significant oversight.

Systemic Toxicity of Smoke Components:MS contains known toxicants, including PM2.5/PM10, aldehydes (e.g., formaldehyde), and polycyclic aromatic hydrocarbons (PAHs). While the study excludes “toxic gaseous compounds,” it provides no data on the safety of identified components (e.g., phthalates, terpenoids) in long-term use. For instance, 邻苯二甲酸酯 (phthalates) are endocrine disruptors linked to metabolic and reproductive disorders, and PAHs are carcinogenic. The absence of toxicity profiling (e.g., hepatic/renal function assays, genotoxicity tests) undermines the safety narrative.

2. Mechanistic Gaps and Incomplete Data Interpretation

Contradictory Findings with MMP12 Upregulation:The study reports that high-dose MS (HMS) significantly upregulates MMP12 mRNA in lung tissue (p<0.05), yet dismisses this as a “potential risk” without mechanistic exploration. MMP12’s role in alveolar wall destruction and fibrosis is well-established; its induction by MS contradicts the claimed “protective effect” on lung injury. The authors must clarify whether this is a dose-dependent effect or an experimental artifact, ideally through immunohistochemistry or functional assays (e.g., ECM degradation markers).

Unclear Dynamics of PPARγ/STAT3 Pathways:While MS upregulates PPARγ and reduces p-STAT3, the study does not address how these changes balance antiviral immunity and inflammatory suppression. For example, PPARγ overactivation may impair macrophage antiviral function, while STAT3 inhibition could compromise viral clearance. Long-term outcomes (e.g., viral persistence, secondary infections) are not evaluated, limiting conclusions about therapeutic windows.

3. Experimental Design Limitations

Lack of Long-Term Exposure Models:The study uses short-term MS exposure (4 days) followed by acute infection, which does not mimic clinical moxibustion practices (often involving repeated sessions). Chronic toxicity endpoints (e.g., lung histology after 28 days, systemic organ damage) are absent, precluding assessment of cumulative risks.

Inadequate Control of Confounding Variables:The study does not specify the combustion conditions of moxa sticks (e.g., temperature, airflow), which profoundly influence smoke composition. Variations in 艾条 (moxa stick) quality (e.g., age of Artemisia argyi, processing methods) may lead to inconsistent results, affecting reproducibility.

Recommendations for Revision

1. Expand Safety Evaluation:

Include chronic toxicity experiments in mice (e.g., 4-week MS exposure without infection) to assess pulmonary fibrosis (e.g., collagen deposition via Masson’s trichrome staining), MMP12 activity, and systemic organ toxicity (liver/kidney function tests).

Characterize MS components using advanced analytical techniques (e.g., GC-MS, HPLC) to identify potential carcinogens/endocrine disruptors, and reference occupational exposure limits (e.g., OSHA standards for formaldehyde, PM2.5).

2. Clarify Mechanistic Paradoxes:

Investigate the temporal and dose-dependent effects of MS on MMP12 expression. Is MMP12 upregulation a late-phase response or specific to high concentrations?

Perform gain/loss-of-function experiments (e.g., PPARγ agonists/antagonists) to dissect its dual role in inflammation and viral clearance.

3. Enhance Clinical Translational Data:

Conduct a retrospective survey of acupuncturists’ respiratory health to correlate MS exposure with chronic pharyngitis or COPD incidence.

Develop low-toxicity MS formulations (e.g., smokeless moxibustion) and compare their efficacy/toxicity with traditional MS in parallel experiments.

4. Revise Discussion Section:

Acknowledge the trade-off between MS’s therapeutic benefits and safety risks, particularly for vulnerable populations (pregnant women, elderly, individuals with preexisting lung disease).

Highlight the need for standardized MS protocols in clinical settings to minimize exposure while preserving efficacy.

Conclusion

This study makes valuable contributions to understanding MS’s anti-influenza mechanisms but lacks critical data on safety and long-term effects. To advance this research toward clinical applicability, the authors must address the identified gaps, particularly regarding MS-induced MMP12 activation and chronic toxicity. With rigorous revisions, the manuscript could provide a more balanced evaluation of MS’s therapeutic potential and risks, thereby guiding responsible use in TCM practice.
