# Supplementary material for: Investigation of the effect and mechanisms of moxa smoke in the treatment of Influenza A Virus (IAV) infection
Source: PLoS One. 2025 Dec 12;20(12):e0337906. doi: 10.1371/journal.pone.0337906 (PMC12700386; doi:10.1371/journal.pone.0337906)
Supplement: Attachment — Submitted filename: Response to Reviewers.docx [file pone.0337906.s005.docx]

Dear editor and reviewers:

We thank all the editors and reviewers for their valuable comments and suggestions. We have carefully revised the manuscript to enhance its clarity and·facilitate the understanding of the readers. Our point-to-point responses are presented in the following." We hope that the revision would satisfactorily" address the comments and conceits of the editors and reviewers.

Below, we have addressed all the additional requirements as outlined in your feedback:

**Comments from the Editor:**

1.Please ensure that your manuscript meets PLOS ONE's style requirements, including those for file naming. The PLOS ONE style templates can be found at

<https://journals.plos.org/plosone/s/file?id=wjVg/PLOSOne_formatting_sample_main_body.pdf> and

[https://journals.plos.org/plosone/s/file?id=ba62/PLOSOne_formatting_sample_title_authors_affiliations.pdf](https://track.editorialmanager.com/CL0/https:%2F%2Fjournals.plos.org%2Fplosone%2Fs%2Ffile%3Fid=ba62%2FPLOSOne_formatting_sample_title_authors_affiliations.pdf/1/010f0197d6a120ce-790f00b5-8f38-4312-bb6d-b0ca33515f91-000000/JAVC_4csN7q4jUE3RdsOz7_zqthiaUk9BV_FfzxKwAI=218)

**Response：**We have carefully revised the manuscript to comply with PLOS ONE’s style requirements, using the provided templates. All files have been renamed according to the journal’s guidelines.

2. Please note that PLOS ONE has specific guidelines on code sharing for submissions in which author-generated code underpins the findings in the manuscript. In these cases, we expect all author-generated code to be made available without restrictions upon publication of the work. Please review our guidelines at <https://journals.plos.org/plosone/s/materials-and-software-sharing#loc-sharing-code> and ensure that your code is shared in a way that follows best practice and facilitates reproducibility and reuse.

**Response**：Our research doesn’t involve any author-generated code.

3. Thank you for stating the following financial disclosure:

“Guangzhou Key Laboratory of Traditional Chinese Medicine for the Prevention and Treatment of Chronic Cough and Dyspnea (Grant Number 2023A03J0226), the double world-class and high-level university discipline collaborative innovation team project of Guangzhou University of Chinese Medicine (Grant Number 2021XK27).”

Please state what role the funders took in the study.  If the funders had no role, please state: "The funders had no role in study design, data collection and analysis, decision to publish, or preparation of the manuscript."

If this statement is not correct you must amend it as needed.

Please include this amended Role of Funder statement in your cover letter; we will change the online submission form on your behalf.

**Response:** We have updated the information regarding fund allocations. This update includes the addition of the fund named ‘Investigation into the Optimal Dosage of Flu Dual-Relief Granules for the Treatment of Viral Pneumonia and the Underlying Mechanisms Involving Energy Metabolism Regulation in Macrophage Polarization (Grant Number 2022B1515230001)’, which covers publication expenses. We have also clarified the role of all the funds, as per your request, in both the manuscript and the cover letter.

4. We note that your Data Availability Statement is currently as follows: All relevant data are within the manuscript and in Supporting Information files.

Please confirm at this time whether or not your submission contains all raw data required to replicate the results of your study. Authors must share the “minimal data set” for their submission. PLOS defines the minimal data set to consist of the data required to replicate all study findings reported in the article, as well as related metadata and methods (<https://journals.plos.org/plosone/s/data-availability#loc-minimal-data-set-definition)>.

For example, authors should submit the following data:

- The values behind the means, standard deviations and other measures reported;

- The values used to build graphs;

- The points extracted from images for analysis.

Authors do not need to submit their entire data set if only a portion of the data was used in the reported study.

If your submission does not contain these data, please either upload them as Supporting Information files or deposit them to a stable, public repository and provide us with the relevant URLs, DOIs, or accession numbers. For a list of recommended repositories, please see <https://journals.plos.org/plosone/s/recommended-repositories>.

If there are ethical or legal restrictions on sharing a de-identified data set, please explain them in detail (e.g., data contain potentially sensitive information, data are owned by a third-party organization, etc.) and who has imposed them (e.g., an ethics committee). Please also provide contact information for a data access committee, ethics committee, or other institutional body to which data requests may be sent. If data are owned by a third party, please indicate how others may request data access.

**Response:** We confirm our submission contains the complete minimal data set required to replicate all study findings. It has been uploaded as Supporting Information files [File names: S2_raw_datasets]

5. Please include a copy of Table 4 which you refer to in your text on page 22. On page 22,

**Response:** "Table 4" was erroneously noted - this has been corrected to "Table 3" throughout the manuscript. The complete Table 3 is now properly included in the main document on page 24.

6. PLOS ONE now requires that authors provide the original uncropped and unadjusted images underlying all blot or gel results reported in a submission’s figures or Supporting Information files. This policy and the journal’s other requirements for blot/gel reporting and figure preparation are described in detail at <https://journals.plos.org/plosone/s/figures#loc-blot-and-gel-reporting-requirements> and <https://journals.plos.org/plosone/s/figures#loc-preparing-figures-from-image-files>. When you submit your revised manuscript, please ensure that your figures adhere fully to these guidelines and provide the original underlying images for all blot or gel data reported in your submission. See the following link for instructions on providing the original image data: <https://journals.plos.org/plosone/s/figures#loc-original-images-for-blots-and-gels>.

**Response：**All original, uncropped blot/gel images have been uploaded as Supporting Information files [File names: S1_raw_images]

**Comments from reviewers：**

**Reviewer #1：**

**Overall Evaluation**

This study explores the antiviral and anti-inflammatory effects of moxa smoke (MS) against H1N1 infection using network pharmacology, molecular docking, and in vivo experiments. The integration of traditional Chinese medicine (TCM) with modern pharmacological approaches is commendable, offering novel insights into MS’s potential therapeutic applications. However, while the research demonstrates scientific rigor in certain aspects, it contains critical gaps, particularly regarding clinical safety implications and mechanistic clarity, which must be addressed for publication.

**Strengths and Innovations**

1. Multidisciplinary Approach to Mechanism DiscoveryThe study employs network pharmacology to systematically identify MS components (52 compounds) and their potential targets (384 proteins), uncovering 16 core targets (e.g., STAT3, PPARγ) via PPI network analysis. This “multi-component, multi-target” framework aligns with TCM’s holistic philosophy and provides a robust basis for understanding MS’s complex actions. The molecular docking validation of stable binding between MS compounds (e.g., Bis-(3,5,5-trimethylhexyl) phthalate) and key targets further strengthens the mechanistic hypothesis.

2. In Vivo Validation of Antiviral and Anti-Inflammatory EffectsUsing a PR8-infected mouse model, the study demonstrates that MS reduces viral load (H1N1NP mRNA), alleviates pulmonary inflammation (e.g., decreased BALF leukocytes, IL-6/TNF-α expression), and modulates PPARγ/STAT3 pathways. These findings provide experimental evidence for MS’s therapeutic potential, particularly its ability to suppress cytokine storms—a critical pathology in severe influenza.

3. Translational Relevance to TCM PracticeBy focusing on MS, a byproduct of moxibustion with historical use in infection prevention, the study bridges traditional practices with modern science. The identification of PPARγ as a key regulator offers a mechanistic explanation for MS’s anti-inflammatory effects, potentially guiding the development of targeted TCM interventions.

**Critical Weaknesses and Safety Concerns**

**1. Inadequate Assessment of Clinical Safety Risks**
Occupational Exposure Risks for Acupuncturists: The manuscript fails to address the chronic toxicity of MS, a major concern for acupuncturist) and patients. Existing evidence (cited in prior reviews) shows that long-term exposure to moxa smoke is associated with increased incidence of chronic pharyngitis, allergic reactions, and potential pulmonary fibrosis. For example, elevated MMP12 expression in MS-treated mice (Table 3) indicates ECM degradation, a hallmark of emphysema and pulmonary fibrosis. The study’s failure to discuss this finding’s clinical relevance constitutes a significant oversight.

Systemic Toxicity of Smoke Components: MS contains known toxicants, including PM2.5/PM10, aldehydes (e.g., formaldehyde), and polycyclic aromatic hydrocarbons (PAHs). While the study excludes “toxic gaseous compounds,” it provides no data on the safety of identified components (e.g., phthalates, terpenoids) in long-term use. For instance, phthalates are endocrine disruptors linked to metabolic and reproductive disorders, and PAHs are carcinogenic. The absence of toxicity profiling (e.g., hepatic/renal function assays, genotoxicity tests) undermines the safety narrative.

**Response：**We appreciate the reviewers' comments. Indeed, prolonged exposure to moxa smoke poses significant occupational hazards for acupuncturists that warrant careful attention. Through a comprehensive literature review and our team's qualitative LC-MS analysis of moxa smoke, we found the compounds such as benzaldehyde, phenol, benzonitrile, and other toxic substances known to cause respiratory irritation, hepatotoxicity, nephrotoxicity, metabolic disruption, and even carcinogenic effects. Additionally, inhalable particulate matter PM10 was detected. In response to the reviewers’ comments, we further investigated the effects of both short-term and long-term moxa smoke exposure on hepatic and renal function, organ mass, pulmonary tissue damage, and collagen deposition. In conjunction with the results of previous clinical studies, it thoroughly evaluates the safety of short-term exposure and the toxicity associated with long-term exposure, as well as the underlying material basis.

These refinements enhance the safety considerations and clinical relevance of the findings, making the research more comprehensive and systematic. Specific results are detailed in the **Recommendations for Revision (Page 9-16)**. The reviewers' suggestion to conduct research on the long-term toxicity of harmful substances in smoke is a valuable recommendation, and we shall progressively undertake such studies in our future work.

**2. Mechanistic Gaps and Incomplete Data Interpretation**

Contradictory Findings with MMP12 Upregulation: The study reports that high-dose MS (HMS) significantly upregulates MMP12 mRNA in lung tissue (p<0.05), yet dismisses this as a “potential risk” without mechanistic exploration. MMP12’s role in alveolar wall destruction and fibrosis is well-established; its induction by MS contradicts the claimed “protective effect” on lung injury. The authors must clarify whether this is a dose-dependent effect or an experimental artifact, ideally through immunohistochemistry or functional assays (e.g., ECM degradation markers).

Unclear Dynamics of PPARγ/STAT3 Pathways: While MS upregulates PPARγ and reduces p-STAT3, the study does not address how these changes balance antiviral immunity and inflammatory suppression. For example, PPARγ overactivation may impair macrophage antiviral function, while STAT3 inhibition could compromise viral clearance. Long-term outcomes (e.g., viral persistence, secondary infections) are not evaluated, limiting conclusions about therapeutic windows.

**Response：** We are grateful for the reviewer’s insightful questions. Elevated MMP12 expression is indeed a significant contributing factor in the development of pulmonary emphysema and fibrosis. However, the research team maintains that this does not contradict the role of MS in suppressing viral replication and mitigating inflammatory storms. Rather, it emphasises the necessity of thoroughly considering its safety profile in clinical applications. This can be achieved through modern scientific techniques, such as purifying the active constituents and removing particulate matter and toxic substances, thereby minimising potential adverse effects. This study, employing network pharmacology methods alongside toxicological research, is well suited to advancing the identification of both therapeutic and toxic components, thereby facilitating further development. In response to the reviewer’s suggestions, this study examined the dose- and time-dependent effects of moxa smoke on MMP12. Combining pulmonary pathology and collagen detection results, we discuss the reasons and correlations between elevated MMP12 levels induced by both short-term and long-term (four weeks) MS exposure, and pulmonary tissue destruction. Relative results and discussion are detailed in the **Recommendations for Revision (Page16)**.

Regarding the PPARγ/STAT3 pathway, we further validated the efficacy of high-dose MS against PR8 viral infection. Additionally, we investigated the effects of PPARγ agonists，and the combined effect of high-dose MS with PPARγ inhibitors. This approach explored the anti-inflammatory mechanism of MS, which depends on PPARγ activation. We discovered that MS exhibits antiviral activity independent of PPARγ, revealing its unique pharmacological property of achieving a balanced effect between anti-inflammatory and antiviral actions despite apparent contradictions. Specific results are detailed in the **Recommendations for Revision (Page17-20)**.

This supplementary data further facilitates a systematic elucidation of the effects of MS on tissue destruction and collagen deposition, clarifies its anti-inflammatory mechanisms, and enhances the depth of the research.

**3. Experimental Design Limitations**

Lack of Long-Term Exposure Models: The study uses short-term MS exposure (4 days) followed by acute infection, which does not mimic clinical moxibustion practices (often involving repeated sessions). Chronic toxicity endpoints (e.g., lung histology after 28 days, systemic organ damage) are absent, precluding assessment of cumulative risks.

Inadequate Control of Confounding Variables:

The study does not specify the combustion conditions of moxa sticks (e.g., temperature, airflow), which profoundly influence smoke composition. Variations in moxa stick quality (e.g., age of Artemisia argyi, processing methods) may lead to inconsistent results, affecting reproducibility.

**Response：** We acknowledge the shortcomings of this study as highlighted by the reviewers. Indeed, the experimental design employed short-term MS exposure (4 days) followed by acute infection, rather than post-infection treatment, which does not fully replicate clinical moxibustion practice. This limitation primarily arises from constraints within the experimental conditions. Our smoke exposure protocol required placing mice from the same treatment group together in a single smoke-exposure chamber three times daily, with the chamber lid opened every 25 minutes within a fume cupboard to ensure ventilation. Administering MS exposure after viral challenge would have significantly increased the risk of aerosol transmission of the virus among groups, thereby compromising the reliability of the experimental results. Consequently, we adopted the approach of administering treatment prior to viral challenge. Fortunately, the beneficial anti-inflammatory and antiviral effects of moxa smoke were observed after only four days of exposure.

Influenza virus infection is inherently a self-limiting disease. Following infection, mice reach peak viral replication and inflammation within 3–5 days, with the virus completely cleared around day 7-10. This pattern mirrors that of human influenza infection. Consequently, our therapeutic strategy prioritises achieving effective control as swiftly as possible. In response to concerns regarding occupational exposure for clinicians and elevated MMP12 levels, we fully concur with the reviewers' observations and have conducted relevant toxicological studies.

Detailed descriptions of the MS preparation and exposure protocols have been revised in the manuscript (lines 172–190) as follows:

Mice were exposed to Moxa smoke from moxa sticks (Chi Fang Laoren brand, manufactured in Qichun County, Hubei Province, China) of different weights. Simply, the raw material for making the sticks is three-year-old Qichun Artemisia argyi grown in Qichun County, Hubei Province, China. The leaves are sun-dried firstly and repeatedly ground to remove coarse and hard impurities, until one kilogram of fine Artemisia argyi floss is obtained from every ten kilograms of dried Artemisia argyi leaves. Then the floss is rolled into moxa sticks of 18mm x 200mm.

In this study, two concentrations of moxa smoke were utilized: low moxa smoke (LMS, 0.3 g moxa stick every 30min, resulting in a PM10 concentration of approximately 1.335 mg/m³) and high moxa smoke (HMS, 0.6 g moxa stick every 30min, resulting in a PM10 concentration of approximately 2.67 mg/m³)[21]. Both PM10 concentrations were lower than the typical concentration of 3.54 mg/m³ found in moxibustion clinics[22]. Different groups of mice were given corresponding concentrations of moxa smoke every day for four days or four weeks.

The exposure was conducted in the ventilation hood. The burned moxa sticks and the mice were placed in a 36-liter plastic chamber (60 x 40 x 15 cm) which is with 3 holes (5mm of the diameter) on the lid to ensure air circulation. Mice were exposed to tobacco smoke three times daily at 9:00 AM, 12:00 PM, and 3:00 PM, with each session lasting 1.5 hours. After every 25 minutes of exposure, the chamber lid was fully opened for 5 minutes to ensure adequate air exchange within the chamber.

Control animals were housed in identical chambers without exposure to moxa smoke. Body weight measurements were recorded at the same time each morning before the initiation of treatment.

The oxygen concentration and temperature within the exposure chamber at different time points following the burning of moxa sticks are as follows:

| Time | 0.3g MS | | 0.6gMS | |
| --- | --- | --- | --- | --- |
|  | O_2_（%） | T（℃） | O_2_（%） | T（℃） |
| 0 min | 20.7 | 22.4 | 20.8 | 22.6 |
| 5 min | 19.9 | 22.6 | 19.9 | 22.8 |
| 10 min | 19.9 | 22.6 | 19.6 | 23 |
| 15 min | 19.9 | 22.6 | 19.6 | 23.4 |
| 20 min | 20.2 | 22.6 | 19.6 | 23.8 |
| 25 min | 20.3 | 22.7 | 19.7 | 23.9 |

**Recommendations for Revision**
**1. Expand Safety Evaluation:**
Include chronic toxicity experiments in mice (e.g., 4-week MS exposure without infection) to assess pulmonary fibrosis (e.g., collagen deposition via Masson’s trichrome staining), MMP12 activity, and systemic organ toxicity (liver/kidney function tests).
Characterize MS components using advanced analytical techniques (e.g., GC-MS, HPLC) to identify potential carcinogens/endocrine disruptors, and reference occupational exposure limits (e.g., OSHA standards for formaldehyde, PM2.5).

**Response：**We have completed a four-week toxicological evaluation of MS exposure in normal mice, which included measurements of lung, liver, kidney, and genital organ weights; pathological examinations; and assessments of serum creatinine, blood urea nitrogen, ALT, AST, and total CO₂ levels. Observations also encompassed Masson's trichrome staining of lung tissue, assessment of pulmonary emphysema formation (MLI index), MMP12 mRNA expression levels, and hydroxyproline content (a collagen metabolite) in lung tissue.

Results indicated that, compared to control mice, lung tissue weight was significantly increased in mice exposed to MS smoke for four weeks. Histopathological examination revealed increased immune cell infiltration within the lung tissue. Serum TCO₂ levels were elevated in mice exposed to a high dose, suggesting that four weeks of MS exposure induced pulmonary inflammation and potentially impaired lung function. High-dose MS inhalation increased kidney weight and renal tubular interstitial space in female mice, with a trend towards elevated serum creatinine (P > 0.05), suggesting mild renal oedema. Additionally, we observed decreased serum ALT and urea levels in MS-exposed mice, although both remained within normal ranges for mice. The AST/ALT ratio remained unchanged. This shift is likely attributable to reduced food intake rather than hepatic dysfunction. The pathology of other organs in MS-exposed mice showed no significant alterations. These findings indicate that four weeks of MS exposure primarily induces pulmonary and renal damage (Figure 10, Table 5 in manuscript).

Four weeks of MS exposure still resulted in elevated MMP12 levels; however, we did not observe the development of pulmonary emphysema. Collagen deposition in the lungs, assessed via Masson’s trichrome staining and hydroxyproline measurement, also showed no significant changes (Figure 11B-D). A possible explanation is that, although MMP12 levels increased, they remained within the range of tissue compensation, thereby failing to cause significant tissue damage or abnormal collagen deposition. Nevertheless, this does not preclude the risk of more severe lung tissue damage arising from prolonged exposure.

To determine whether short-term exposure to MS affects liver and kidney function in virus-infected mice, or alters pulmonary collagen levels or causes pulmonary emphysema due to elevated MMP12, we examined mice treated with MS for four days prior to viral infection. Assessments included Masson's trichrome staining, hydroxyproline levels (a collagen metabolite), and pulmonary emphysema formation (MLI index). Results indicated that short-term MS exposure did not cause abnormal liver (AST levels in PR8-infected mice exceed the normal range, which is attributed to inflammation induced by the infection. MS alleviated the inflammation and AST level.) or kidney function, nor CO₂ retention. Although MMP12 levels in lung tissue were significantly elevated compared to virus-infected mice (Table 3), no evidence of collagen deposition or pulmonary emphysema formation was observed. These findings suggest the safety of short-term MS exposure (Figure 11 B-D).

The group previously employed chloroform to extract constituents from MS, subsequently characterising the components using GC-MS. The total ion chromatogram is as follows:

The results revealed not only potential active compounds such as guaiacol, α-terpineol, 4-terpineol, and camphor, but also toxic substances including aldehydes, phenols, and polycyclic aromatic hydrocarbons. According to the literature, benzaldehyde, benzonitrile, 1,1,2,2-tetrachloroethane, and phenol not only cause respiratory tract irritation leading to respiratory system damage, but also exhibit hepatotoxicity, nephrotoxicity, metabolic disruption, and even carcinogenic effects. These factors account for the toxic effects observed following four weeks of MS exposure. Interestingly, however, existing clinical studie [Evid Based Complement Alternat Med. 2019 Sep 16:2019:4873235.][J Tradit Chin Med. 2018 Feb;38(1):132-138.] rarely report organ damage as severe as that observed in the animal study, despite longer exposure durations. We propose that this discrepancy may arise from the use of enclosed-space exposure protocols in animal studies, which are employed to maintain controllable smoke concentrations. Such conditions exacerbate the production of harmful substances from the incomplete combustion of moxa sticks, in contrast to the well-ventilated environments typical of clinical consultation rooms [Int J Environ Res Public Health. 2022 Mar 25;19(7):3944. doi: [10.3390/ijerph19073944](https://doi.org/10.3390/ijerph19073944)].

Additionally, the research team monitored PM10 levels within the exposure chamber during the experiment. The PM10 concentration in the high-dose group ranged from approximately 2.51 to 3 mg/m³. Currently, no international occupational exposure standards exist for inhalable particulate matter. However, it is evident from our study that the PM10 levels (approximately 500 μg/m³ over 24 hours) exceeded China’s established standard for normal outdoor PM10 (150 μg/m³ over 24 hours). Consequently, prolonged exposure poses a risk.

Consequently, refining animal experimental methods could yield findings more closely aligned with clinical practice. In clinical applications, attention should be paid to ventilation standards in consultation rooms, alongside increased scrutiny of safety assessments for moxa smoke exposure.

We added the descriptions in the manuscript as follows( Line 524-555, Line 562-576, Line 680-705 in manuscript):

**Results**

9. Safety assessment of MS

MS exposure for 4 days in H1N1-infected mice

Table 4 presents the serum biochemical parameters of H1N1 infected mice subjected to moxa smoke exposure for a duration of 4 days. Analysis of the blood biochemical markers revealed that, compared to the PR8 group, both the LMS and HMS group exhibited reductions in ALT and AST levels. The LMS also reduced Urea of the H1N1 infected mice.

MS exposure for 4 weeks in normal mice

Table 5 presents the blood biochemical parameters of mice subjected to moxa smoke exposure for a duration of 4 weeks. Analysis of the blood biochemical markers revealed that, relative to the sham group, the LMS group exhibited reductions in AST and Urea levels in male mice. And increased the TCO2 in male mice. In contrast, the HMS group demonstrated decreased Urea concentrations, alongside elevated TCO2 levels in both male and female mice, when compared to the sham group. HMS also reduced the level of ALT in male mice compared to sham mice.

Figure 10A showed the organ weight ratio of sham mice after MS exposure. Mice exposed to LMS and HMS both showed increase in the ratios of lung /body weight. Female mice exposed to HMS exhibited an increased kidney-to-body weight ratio.

Histopathological examination (Fig 10B) showed that (1) Compared to control mice, more macrophages were observed in lungs exposed with both LMS and HMS, and the alveolar septum structure was normal. (2) Compared to control mice, renal tubular epithelial cells were completely, there were no obviously granules and vacuolar degeneration. However, in female mice which exposed to HMS, there were slightly interstitial edema. (3) The liver, kidney, testis, and ovary tissues after four weeks of MS exposure revealed no discernible pathological abnormalities compared to the sham controls.

**Table 4. Serum biochemistry parameters of** **H1N1-infected mice treated with inhaled MS for 4 days**

| **Serum biochemistry** | **PR8** | **PR8+LMS** | **PR8+HMS** |
| --- | --- | --- | --- |
| **ALT/U·L⁻¹** | 39.14±2.0 | 26.38±0.68**** | 27.88±0.74**** |
| **AST/U·L⁻¹** | 116.4±9.26 | 83.75±3.99** | 72.75±4.2*** |
| **AST/ALT** | 3.27±0.30 | 3.2±0.19 | 2.613±0.18 |
| **Urea/mol·L⁻¹** | 6.46±0.39 | 4.838±0.24** | 6.05±0.43 |
| **Cr/μmol·L⁻¹** | 5.86±0.26 | 5.5±0.5 | 5±0.27 |
| **TCO2/mmol·L⁻¹** | 20.71±0.74 | 20.53±0.47 | 20.98±0.31 |

Data are expressed as mean ± S.E.M for n = 7-9 female mice per treatment group. One-way ANOVA was performed to assess statistical significance; ***p*<0.01,****p*<0.001, *****p*<0.0001.

**Table 5.** **Serum biochemistry parameters of mice treated with inhaled MS for 4 weeks**

| **Serum biochemistry** | **Gender** | **sham** | **LMS** | **HMS** |
| --- | --- | --- | --- | --- |
| **ALT/U·L⁻¹** | Female | 29±0.58 | 26.33±1.2 | 25.75±1.11 |
|  | Male | 32.8±3.09 | 28.8±1.02 | 25.25±1.03* |
| **AST/U·L⁻¹** | Female | 78.25±4.25 | 82.67±6.69 | 73.5±1.76 |
|  | Male | 78.4±1.17 | 65.4±3.75* | 70.75±6.63 |
| **AST/ALT** | Female | 2.7±0.11 | 3.13±0.15 | 2.85±0.17 |
|  | Male | 2.44±0.19 | 2.26±0.12 | 2.78±0.18 |
| **Urea/mol·L⁻¹** | Female | 6.43±0.38 | 5.33±0.23 | 4.95±0.26** |
|  | Male | 8.4±0.39 | 5.64±0.22**** | 5.38±0.15**** |
| **Cr/μmol·L⁻¹** | Female | 6.48±0.67 | 6.67±0.33 | 7.75±0.25 |
|  | Male | 5.98±0.56 | 5.18±0.21 | 4.95±0.03 |
| **TCO2/mmol·L⁻¹** | Female | 17.3±0.69 | 18.93±0.07 | 18.85±0.35* |
|  | Male | 18.2±0.43 | 19.88±0.35* | 21.03±0.44*** |

n=5 for male，n=5 for female. Data are expressed as mean ± S.E.M. One-way ANOVA was performed to assess statistical significance; **p*<0.05, ***p*<0.01, ****p*<0.001, *****p*<0.0001.

10. MS promotes MMP12 expression, but has not yet led to the development of emphysema or abnormal collagen deposition in 4 weeks

The MMP12 mRNA in lung tissue were increased after both 4-day MS exposure and 4-week MS exposure compared to sham mice. Moreover, compared to the mice exposed to MS for 4 days, the expression level of MMP12 in the lung tissue of mice exposed to MS for 4 weeks is higher. This result indicates that the increase in lung MMP12 levels induced by MS is time-dependent (Fig 11A).

To clarify whether short-term exposure to MS causes abnormal lung collagen levels or emphysema formation in influenza virus-infected mice and normal mice with long-term exposure to MS, we measured lung collagen level by hydroxyproline measurement and Masson's trichrome staining, and the emphysema formation (MLI index) was also detected in influenza-infected mice exposed to MS for 4 days and normal mice exposed to MS for 4 weeks. The results demonstrated that the collagen level and MLI index didn’t change after 4-day or 4-week exposure of MS (Fig 11 B-D).

Discussion

Although studies indicate that exposure to MS does not significantly affect the health of physicians[79-80], the presence of harmful substances in moxa smoke[81] and evidence from animal studies suggesting that it impairs respiratory function[82] make it imperative to investigate its safety profile. This study first examined biochemical parameters in influenza-infected mice following short-term exposure to moxa smoke. The results indicated no apparent abnormalities in liver or kidney function. Although MMP12 expression increased in the lungs, no emphysema or abnormal collagen deposition was observed, nor was carbon dioxide retention detected in the blood. In contrast, normal mice exposed to moxa smoke for four weeks exhibited increased lung tissue weight, heightened inflammatory cell infiltration in the lungs, elevated pulmonary MMP12 expression (without inducing pulmonary emphysema or changes in collagen deposition) and mild oedema of renal tissue in the high-dose moxa smoke exposure group. These findings indicate that short-term exposure to moxa smoke is safe, but prolonged exposure causes significant damage to the lungs and renal function. Furthermore, compounds in moxa smoke, such as phenol, benzonitrile, and 1,1,2,2-tetrachloroethane, can cause respiratory system damage, alongside hepatotoxicity, nephrotoxicity, metabolic disruption, and carcinogenic risks[83-85].

Furthermore, animal studies often burn moxa sticks in relatively confined spaces to control smoke concentration, which paradoxically exacerbates incomplete combustion. The resulting increase in harmful substances heightens toxicity, creating a discrepancy with real-world clinical settings.

**2. Clarify Mechanistic Paradoxes:**
Investigate the temporal and dose-dependent effects of MS on MMP12 expression. Is MMP12 upregulation a late-phase response or specific to high concentrations?
Perform gain/loss-of-function experiments (e.g., PPARγ agonists/antagonists) to dissect its dual role in inflammation and viral clearance.

**Response：**

1**）**Following the reviewers' suggestions, we assessed MMP12 mRNA levels in mouse lung tissue after exposure to varying doses of cigarette smoke at 4 days and 4 weeks (Figure 11A). We observed that the elevation of MMP12 exhibited both dose-dependent and time-dependent characteristics. This indicates a causal relationship between MMP12 expression and smoke inhalation, demonstrating cumulative effects rather than a late-phase response. Fortunately, this reaction does not cause lung tissue damage or excessive collagen deposition within the short-term exposure period, but moxa smoke inhalation maybe unsuitable as a treatment modality for prolonged exposure.

2) To determine whether moxa smoke reduces inflammation and viral replication by activating PPARγ, we further investigated the effects of combining a PPARγ inhibitor with high-dose moxa smoke and the PPARγ agonist alone. The results revealed that high-dose moxa smoke and PPARγ agonists reduced cellular counts in airway lavage fluid, whereas the combination of high-dose moxa smoke with PPARγ inhibitors failed to effectively decrease cellular levels in the lavage fluid. These findings are consistent with previous reports indicating that PPARγ agonists can suppress excessive inflammatory responses induced by influenza viruses.

The question of whether PPARγ agonists can inhibit viral replication remains a matter of debate in the existing literature. Hongbo Zhang et al. reported that PPARγ agonists can suppress influenza virus replication [Front Immunol. 2022 Aug 25:13:958801.]; however, other studies have found no effect of PPARγ agonists on influenza viral load [Front Immunol. 2022 Aug 16:13:968336.] [PLoS One. 2010 Oct 12;5(10): e13251]. Our current findings are consistent with the latter ones, showing no inhibitory effect of PPARγ agonists on viral replication.

Nevertheless, our findings indicate that both high-dose exposure to MS and combined exposure to high-dose MS with PPARγ inhibitors effectively reduce viral load in mouse lungs. This suggests the presence of additional antiviral substances within MS. Based on a review of the literature, we discuss potential antiviral compounds in MS in the Discussion section on Line 599-603 “In the current study, 52 components were identified in moxa smoke. There are many essential oil components in moxa smoke, including camphor[35-38], 4-Terpineol[37,39-40], L-alpha-terpineol[39], α,β-thujone[41-42], D-limonene[41,43-44], caryophyllene oxide[45^-^46], β-caryophyllene[44-47], 2,3-dehydro-1,8-cineole[49], and 1,8-cineole[50-55], which have been shown to possess antiviral effects.”Moreover, we observed that the combination of MS with PPARγ inhibitors appeared to exert stronger antiviral effects and demonstrated a greater capacity to regulate pulmonary inflammatory factors. This indicates that while the PPARγ inhibitor enabled MS to suppress influenza-induced inflammatory cell recruitment, it simultaneously impaired the smoke’s viral clearance capacity. Conversely, PPARγ inhibition enhanced the smoke’s antiviral clearance effect but increased inflammatory cell recruitment. Consequently, the antiviral and anti-inflammatory effects of MS exhibit a trade-off. It reduces the viral load ( less than MS+ inhibitor), reduces pulmonary tissue inflammation (less than MS+ inhibitor), and simultaneously diminishes immune cell recruitment (more effectively than MS+ inhibitor).

According to the literature, PPARγ agonists can inhibit STAT3 activation; however, this effect was not observed in the present study. The reason may be due to the presence of the influenza virus, which counteracts the inhibitory effect of PPARγ on STAT3 activation. Under conditions of viral suppression, p-STAT3 levels in the moxa smoke plus PPARγ inhibitor group were significantly higher than in the moxa smoke-only group. This indicates that inhibition of STAT3 phosphorylation by activation of PPARγ is one mechanism of MS. However, cytokine alterations appeared to correlate with viral load trends and were unaffected by p-STAT3 levels. This indicates that IL-6 production in virus-infected epithelial cells does not primarily depend on p-STAT3 phosphorylation; rather, Toll-like receptors, NF-κB, NLRP3, MAPK, and other pathways maybe also involved in this complex mechanism. [J Thorac Dis. 2013 Aug;5(Suppl 2): S132–S141.]

We have added the relative description and explanation in Result part and Discussion part as fllowing (Line 502-516,656-680 in manuscript):

Result

8. MS alleviates the recruitment of pro-inflammatory cells by activation of PPARG in H1N1-infected mice

To determine whether MS alleviates airway inflammation and reduces viral load via PPARG activation, we compared the effects of HMS with the PPARG agonist pioglitazone, as well as HMS combined with the PPARG inhibitor GW9662, in H1N1-infected mice.

The results showed that, compared with the model mice, HMS treatment decreased the population of BALF cells, especially neutrophils and lymphocytes (Fig 9A), and reduced the mRNA expression of NP, IL-6, TNF-α, and IL-1β (Fig 9B) in the lung tissue of H1N1-infected mice, consistent with previous findings. When the mice were given GW9662 prior to HMS treatment, the cell population in the BALF, NP, and cytokines were reduced compared to the model mice; however, the number of BALF cells was higher than in mice treated with HMS alone, without GW9662 (Fig 9A and B). Pioglitazone treatment alone reduced the population of BALF cells but did not affect NP or cytokine levels in model mice. Regarding STAT3 phosphorylation, neither pioglitazone nor GW9662 combined with HMS reduced the level of p-STAT3 (Fig 9C).

**Discussion**

Does MS exert its anti-inflammatory and antiviral effects via PPARγ? Our experimental findings indicate that when PPARγ is inhibited by GW9662, the inhibitory effect of moxa smoke on the recruitment of immune cells to the lungs of H1N1-infected mice is markedly diminished, particularly with regard to neutrophils and lymphocytes. This demonstrates that moxa smoke reduces the infiltration of inflammatory cells in the lungs of H1N1-infected mice, and that this effect is dependent on PPARγ.

Interestingly, however, PPARγ agonists failed to reduce the viral load in mouse lungs, whereas moxa smoke significantly decreased the viral load in lung tissue. Following inhibition by PPARγ antagonists, the suppression of viral replication by moxa smoke appeared more pronounced than with moxa smoke alone. This indicates that the reduction in pulmonary viral load caused by moxa smoke does not depend on PPARγ. Conversely, inhibiting PPARγ enhances the viral clearance effect of moxa smoke. The reason may be that the suppression of immune cell recruitment by PPARγ activation partially impedes influenza virus clearance. As previously discussed, certain components within moxa smoke exhibit antiviral activity independent of PPARγ. This explains why moxa smoke exerts a stronger inhibitory effect on immune cells than PPARγ agonists, and why the effect of MS on reducing pulmonary immune cells exists even when PPARγ is inhibit.

Previous literatures indicates that activation of PPARγ can inhibit STAT3 phosphorylation[77-78]. In this study, PPARγ agonists did not markedly reduce p-STAT3 levels. This is because influenza virus infection elevates p-STAT3 levels, thereby masking the inhibitory effect of PPARγ agonists on p-STAT3. When the viral load decreases, inhibiting PPARγ increases p-STAT3 levels; hence, p-STAT3 levels in the moxa smoke + GW9662 group were higher than those in the moxa smoke group. However, according to our results, the primary inflammatory cytokines—IL-6, TNF-α, and IL-1β—appear to correspond with the level of viral load and are not primarily regulated by p-STAT3.

**3. Enhance Clinical Translational Data:**
Conduct a retrospective survey of acupuncturists’ respiratory health to correlate MS exposure with chronic pharyngitis or COPD incidence.
Develop low-toxicity MS formulations (e.g., smokeless moxibustion) and compare their efficacy/toxicity with traditional MS in parallel experiments.

**Response：**We sincerely appreciate the reviewer’s valuable and insightful comments. We fully concur with the reviewer’s concerns regarding the long-term safety of drug use, as safety evaluation is an indispensable component of a drug’s clinical translation pathway.

However, the primary objective of this study was to elucidate the efficacy and mechanism of action of MS in treating influenza virus infection, which falls within the scope of preclinical research. In addition, we have completed a four-week toxicological assessment in accordance with the reviewer’s suggestions. So the clinical investigation into the impact of moxa smoke on the incidence of chronic obstructive pulmonary disease and pharyngitis seems to beyond the primary focus of this study.

The development of low-toxicity moxibustion preparations represents a highly promising area of research. However, achieving this goal involves complex procedures and requires considerable time, which cannot be accomplished through a single experimental study. Our current study constitutes the initial step in this direction, demonstrating the therapeutic efficacy and fundamental mechanisms of MS to pave the way for the development of low-toxicity moxibustion preparations. We shall continue our efforts along this trajectory in subsequent work.

**4. Revise Discussion Section:**
Acknowledge the trade-off between MS’s therapeutic benefits and safety risks, particularly for vulnerable populations (pregnant women, elderly, individuals with preexisting lung disease).
Highlight the need for standardized MS protocols in clinical settings to minimize exposure while preserving efficacy.
**Response**：We have thoroughly discussed the potential toxic effects and toxic substances in MS, supplementing our recommendations regarding suitable user groups and the suggestion to establish exposure control standards.

The details are added in discussion as following(Line 696-705 in manuscript):

Based on the findings of this study, short-term exposure to moxa smoke for the treatment of influenza virus is recommended as both effective and safe. However, prolonged exposure should be avoided wherever possible, with particular restrictions for the elderly, children, and individuals with underlying medical conditions.

This underscores that, in clinical moxa smoke therapy, alongside controlling exposure duration, attention must be paid to environmental ventilation to minimise incomplete combustion. It is also recommended that occupational exposure standards for moxa smoke be established.

**Reviewer #2：**

1. In page 9, line 182-186. The moxa smoker exposure experiment is low moxa smoke (LMS, resulting in a PM10 concentration of approximately 1.335 mg/m³) and high moxa smoke (HMS, resulting in a PM10 concentration of approximately 2.67 mg/m³)21. Both PM10 concentrations were lower than the typical concentration of 3.54 mg/m³ found in moxibustion clinics. But the dosage conversion isn’t correct simply by same unit (mg/m³) for animal and human. As common view, we usually use body surface area method to calculate drug dosage between different species. So in Figure 5, we could find the body weight loss of LMS+PR8 group and HMS+PR8 group reduced seriously compared MEM+sham group from day 2-4 before H1N1 PR8 infection. The author needs to explain these moxa smoke exposure concentrations and also need more data to prove its safety in this dosage.

**Response：**We are most grateful for the reviewer’s comments; the question is very interesting. Our selection of exposure concentrations was primarily based on two considerations: firstly, we adopted the approach used in COPD mouse models, aligning cigarette exposure concentrations as closely as possible with those experienced by adult smokers to ensure clinical relevance. Secondly, excessively high smoke concentrations would cause a sharp decline in oxygen levels within the exposure chamber. Consequently, after repeated experimentation, we selected an exposure concentration lower than that reported in clinical moxibustion treatment rooms and observed the effects of moxa smoke.

Regarding the rationale for the first approach, we contend that our research aims to alleviate pulmonary inflammation via smoke inhalation into the lungs. This differs significantly from oral medications, which reach the target organs via the digestive tract-metabolism- blood circulation. The method of converting drug dosages for humans and animals based on body surface area primarily relies on the pharmacokinetic differences between humans and animals. Our administration method delivers the drug directly to pulmonary cells. Therefore, ensuring concentrations that approximate human exposure appears more reasonable, regardless of whether the cells are of human or animal origin.

However, we previously overlooked a critical issue: toxicity to organs beyond the lungs. Inhaled toxic substances may damage other organs via the pulmonary circulation. Consequently, concentrations considered relevant to humans are excessively high for mice. This may explain why clinical studies, despite longer exposure durations than animal studies, rarely report the pronounced weight loss, hepatic dysfunction, and renal impairment observed in animal experiments.

We have completed a four-week toxicological evaluation of MS exposure in normal mice, which included measurements of lung, liver, kidney, and genital organ weights; pathological examinations; and assessments of serum creatinine, blood urea nitrogen, ALT, AST, and total CO₂ levels. Observations also encompassed Masson's trichrome staining of lung tissue, assessment of pulmonary emphysema formation (MLI index), MMP12 mRNA expression levels, and hydroxyproline content (a collagen metabolite) in lung tissue.

Results indicated that, compared to control mice, lung tissue weight was significantly increased in mice exposed to MS smoke for four weeks. Histopathological examination revealed increased immune cell infiltration within the lung tissue. Serum TCO₂ levels were elevated in mice exposed to a high dose, suggesting that four weeks of MS exposure induced pulmonary inflammation and potentially impaired lung function. High-dose MS inhalation increased kidney weight and renal tubular interstitial space in female mice, with a trend towards elevated serum creatinine (P > 0.05), suggesting mild renal oedema. Additionally, we observed decreased serum ALT and urea levels in MS-exposed mice, although both remained within normal ranges for mice. The AST/ALT ratio remained unchanged. This shift is likely attributable to reduced food intake rather than hepatic dysfunction. The pathology of other organs in MS-exposed mice showed no significant alterations. These findings indicate that four weeks of MS exposure primarily induces pulmonary and renal damage (Figure 10, Table 5 in manuscript).

Four weeks of MS exposure still resulted in elevated MMP12 levels; however, we did not observe the development of pulmonary emphysema. Collagen deposition in the lungs, assessed via Masson’s trichrome staining and hydroxyproline measurement, also showed no significant changes (Figure 11B-D). A possible explanation is that, although MMP12 levels increased, they remained within the range of tissue compensation, thereby failing to cause significant tissue damage or abnormal collagen deposition. Nevertheless, this does not preclude the risk of more severe lung tissue damage arising from prolonged exposure.

To determine whether short-term exposure to MS affects liver and kidney function in virus-infected mice, or alters pulmonary collagen levels or causes pulmonary emphysema due to elevated MMP12, we examined mice treated with MS for four days prior to viral infection. Assessments included Masson's trichrome staining, hydroxyproline levels (a collagen metabolite), and pulmonary emphysema formation (MLI index). Results indicated that short-term MS exposure did not cause abnormal liver (AST levels in PR8-infected mice exceed the normal range, which is attributed to inflammation induced by the infection. MS alleviated the inflammation and AST level.) or kidney function, nor CO₂ retention. Although MMP12 levels in lung tissue were significantly elevated compared to virus-infected mice (Table 3), no evidence of collagen deposition or pulmonary emphysema formation was observed. These findings suggest the safety of short-term MS exposure (Figure 11 B-D).

The group previously employed chloroform to extract constituents from MS, subsequently characterising the components using GC-MS. The total ion chromatogram is as follows:

The results revealed not only potential active compounds such as guaiacol, α-terpineol, 4-terpineol, and camphor, but also toxic substances including aldehydes, phenols, and polycyclic aromatic hydrocarbons. According to the literature, benzaldehyde, benzonitrile, 1,1,2,2-tetrachloroethane, and phenol not only cause respiratory tract irritation leading to respiratory system damage, but also exhibit hepatotoxicity, nephrotoxicity, metabolic disruption, and even carcinogenic effects. These factors account for the toxic effects observed following four weeks of MS exposure. Interestingly, however, existing clinical studie [Evid Based Complement Alternat Med. 2019 Sep 16:2019:4873235.][J Tradit Chin Med. 2018 Feb;38(1):132-138.] rarely report organ damage as severe as that observed in the animal study, despite longer exposure durations. We propose that this discrepancy may arise from the use of enclosed-space exposure protocols in animal studies, which are employed to maintain controllable smoke concentrations. Such conditions exacerbate the production of harmful substances from the incomplete combustion of moxa sticks, in contrast to the well-ventilated environments typical of clinical consultation rooms [Int J Environ Res Public Health. 2022 Mar 25;19(7):3944. doi: [10.3390/ijerph19073944](https://doi.org/10.3390/ijerph19073944)].

Additionally, the research team monitored PM10 levels within the exposure chamber during the experiment. The PM10 concentration in the high-dose group ranged from approximately 2.51 to 3 mg/m³. Currently, no international occupational exposure standards exist for inhalable particulate matter. However, it is evident from our study that the PM10 levels (approximately 500 μg/m³ over 24 hours) exceeded China’s established standard for normal outdoor PM10 (150 μg/m³ over 24 hours). Consequently, prolonged exposure poses a risk.

Consequently, refining animal experimental methods could yield findings more closely aligned with clinical practice. In clinical applications, attention should be paid to ventilation standards in consultation rooms, alongside increased scrutiny of safety assessments for moxa smoke exposure.

We added the descriptions in the manuscript as follows( Line 524-555, Line 562-576, Line 680-705 in manuscript):

**Results**

9. Safety assessment of MS

MS exposure for 4 days in H1N1-infected mice

Table 4 presents the serum biochemical parameters of H1N1 infected mice subjected to moxa smoke exposure for a duration of 4 days. Analysis of the blood biochemical markers revealed that, compared to the PR8 group, both the LMS and HMS group exhibited reductions in ALT and AST levels. The LMS also reduced Urea of the H1N1 infected mice.

MS exposure for 4 weeks in normal mice

Table 5 presents the blood biochemical parameters of mice subjected to moxa smoke exposure for a duration of 4 weeks. Analysis of the blood biochemical markers revealed that, relative to the sham group, the LMS group exhibited reductions in AST and Urea levels in male mice. And increased the TCO2 in male mice. In contrast, the HMS group demonstrated decreased Urea concentrations, alongside elevated TCO2 levels in both male and female mice, when compared to the sham group. HMS also reduced the level of ALT in male mice compared to sham mice.

Figure 10A showed the organ weight ratio of sham mice after MS exposure. Mice exposed to LMS and HMS both showed increase in the ratios of lung /body weight. Female mice exposed to HMS exhibited an increased kidney-to-body weight ratio.

Histopathological examination (Fig 10B) showed that (1) Compared to control mice, more macrophages were observed in lungs exposed with both LMS and HMS, and the alveolar septum structure was normal. (2) Compared to control mice, renal tubular epithelial cells were completely, there were no obviously granules and vacuolar degeneration. However, in female mice which exposed to HMS, there were slightly interstitial edema. (3) The liver, kidney, testis, and ovary tissues after four weeks of MS exposure revealed no discernible pathological abnormalities compared to the sham controls.

**Table 4. Serum biochemistry parameters of H1N1-infected mice treated with inhaled MS for 4 days**

| **Serum biochemistry** | **PR8** | **PR8+LMS** | **PR8+HMS** |
| --- | --- | --- | --- |
| **ALT/U·L⁻¹** | 39.14±2.0 | 26.38±0.68**** | 27.88±0.74**** |
| **AST/U·L⁻¹** | 116.4±9.26 | 83.75±3.99** | 72.75±4.2*** |
| **AST/ALT** | 3.27±0.30 | 3.2±0.19 | 2.613±0.18 |
| **Urea/mol·L⁻¹** | 6.46±0.39 | 4.838±0.24** | 6.05±0.43 |
| **Cr/μmol·L⁻¹** | 5.86±0.26 | 5.5±0.5 | 5±0.27 |
| **TCO2/mmol·L⁻¹** | 20.71±0.74 | 20.53±0.47 | 20.98±0.31 |

Data are expressed as mean ± S.E.M for n = 7-9 female mice per treatment group. One-way ANOVA was performed to assess statistical significance; ***p*<0.01,****p*<0.001, *****p*<0.0001.

**Table 5. Serum biochemistry parameters of mice treated with inhaled MS for 4 weeks**

| **Serum biochemistry** | **Gender** | **sham** | **LMS** | **HMS** |
| --- | --- | --- | --- | --- |
| **ALT/U·L⁻¹** | Female | 29±0.58 | 26.33±1.2 | 25.75±1.11 |
|  | Male | 32.8±3.09 | 28.8±1.02 | 25.25±1.03* |
| **AST/U·L⁻¹** | Female | 78.25±4.25 | 82.67±6.69 | 73.5±1.76 |
|  | Male | 78.4±1.17 | 65.4±3.75* | 70.75±6.63 |
| **AST/ALT** | Female | 2.7±0.11 | 3.13±0.15 | 2.85±0.17 |
|  | Male | 2.44±0.19 | 2.26±0.12 | 2.78±0.18 |
| **Urea/mol·L⁻¹** | Female | 6.43±0.38 | 5.33±0.23 | 4.95±0.26** |
|  | Male | 8.4±0.39 | 5.64±0.22**** | 5.38±0.15**** |
| **Cr/μmol·L⁻¹** | Female | 6.48±0.67 | 6.67±0.33 | 7.75±0.25 |
|  | Male | 5.98±0.56 | 5.18±0.21 | 4.95±0.03 |
| **TCO2/mmol·L⁻¹** | Female | 17.3±0.69 | 18.93±0.07 | 18.85±0.35* |
|  | Male | 18.2±0.43 | 19.88±0.35* | 21.03±0.44*** |

n=5 for male，n=5 for female. Data are expressed as mean ± S.E.M. One-way ANOVA was performed to assess statistical significance; **p*<0.05, ***p*<0.01, ****p*<0.001, *****p*<0.0001.

10. MS promotes MMP12 expression, but has not yet led to the development of emphysema or abnormal collagen deposition in 4 weeks

The MMP12 mRNA in lung tissue were increased after both 4-day MS exposure and 4-week MS exposure compared to sham mice. Moreover, compared to the mice exposed to MS for 4 days, the expression level of MMP12 in the lung tissue of mice exposed to MS for 4 weeks is higher. This result indicates that the increase in lung MMP12 levels induced by MS is time-dependent (Fig 11A).

To clarify whether short-term exposure to MS causes abnormal lung collagen levels or emphysema formation in influenza virus-infected mice and normal mice with long-term exposure to MS, we measured lung collagen level by hydroxyproline measurement and Masson's trichrome staining, and the emphysema formation (MLI index) was also detected in influenza-infected mice exposed to MS for 4 days and normal mice exposed to MS for 4 weeks. The results demonstrated that the collagen level and MLI index didn’t change after 4-day or 4-week exposure of MS (Fig 11 B-D).

Discussion

Although studies indicate that exposure to MS does not significantly affect the health of physicians[79-80], the presence of harmful substances in moxa smoke[81] and evidence from animal studies suggesting that it impairs respiratory function[82] make it imperative to investigate its safety profile. This study first examined biochemical parameters in influenza-infected mice following short-term exposure to moxa smoke. The results indicated no apparent abnormalities in liver or kidney function. Although MMP12 expression increased in the lungs, no emphysema or abnormal collagen deposition was observed, nor was carbon dioxide retention detected in the blood. In contrast, normal mice exposed to moxa smoke for four weeks exhibited increased lung tissue weight, heightened inflammatory cell infiltration in the lungs, elevated pulmonary MMP12 expression (without inducing pulmonary emphysema or changes in collagen deposition) and mild oedema of renal tissue in the high-dose moxa smoke exposure group. These findings indicate that short-term exposure to moxa smoke is safe, but prolonged exposure causes significant damage to the lungs and renal function. Furthermore, compounds in moxa smoke, such as phenol, benzonitrile, and 1,1,2,2-tetrachloroethane, can cause respiratory system damage, alongside hepatotoxicity, nephrotoxicity, metabolic disruption, and carcinogenic risks[83-85].

Furthermore, animal studies often burn moxa sticks in relatively confined spaces to control smoke concentration, which paradoxically exacerbates incomplete combustion. The resulting increase in harmful substances heightens toxicity, creating a discrepancy with real-world clinical settings.

2. In page 23, H1N1 NP gene of MEM+sham group is not correct, it is normal mice, it’s impossible to have H1N1 NP gene. So, these data need to calculate again.

**Response：**We appreciate the reviewer's attention to methodological details. We employ the 2 ^(-ΔΔCt)^ method to calculate the relative copy number of NP expression across groups. The specific algorithm is as follows: ΔCt = NP cycle value – internal reference cycle value; ΔΔCt = ΔCt of the sample minus the average ΔCt of the MEM+sham (control) group. Consequently, for the MEM+sham group, ΔΔCt fluctuates around zero, while 2^(-ΔΔCt)^ fluctuates around one. This algorithm represents the most commonly employed calculation method in real-time PCR, reflecting the relative numerical values of other groups compared to the control group.

3. Did author analysis the number of inflammatory cells in BALF with flow cytometry? If this, the author need provide more experimental information with antibody, company name, experimental condition etc.

**Response：**We did not analyse the number of inflammatory cells in BALF using flow cytometry. Instead, we employed smear preparation combined with histopathological staining, which are consistently used in our laboratory [Clin Sci (Lond). 2019 Feb 18;133(4):551-564.][ Phytomedicine. 2024 Apr: 126:155273.]. We have rewrite the methodology section which provides a more detailed description of this technique on Line 215-220 in the manuscript as following (Line214-221 in manuscript) : Following the collection of bronchoalveolar lavage fluid (BALF), the counting of nucleated cells was performed using a fluorescence microscope after the cells were stained with AO/EB. Subsequently, cytospins were prepared, and the cells were fixed and stained with Epredia Shandon Kwik-Diff Stains (Richard-Allan Scientific, Runcorn, England, United Kingdom). Then the cell differentials were assessed based on their morphological characteristics [25–26]. The cell count results were calculated using the following formula:

Final absolute cell count = Total number of cells × Percentage of each cell type

We also add the represented image in the manuscript as following :

4. Author need to check style of reference, English expression carefully in this manuscript.

**Response:** We have carefully reviewed and standardized the reference formatting in accordance with the journal's guidelines and polished English expression using an online AI gramma checking system. https://wordvice.ai/cn

5. The image resolution is insufficient, need to improve.

**Response:** We have replaced all figures with 600 dpi TIFF files, added scale bars to microscopy images, and confirmed the colour depth as 24-bit RGB for graphs and 16-bit greyscale for blots, in accordance with the journal's request.

We deeply appreciate the reviewers' attention to detail which has improved our manuscript's accuracy. Please find the corrected version with tracked changes and clean copies attached. We hope that the revised manuscript now meets PLOS ONE’s standards and look forward to your favorable consideration. Please let us know if any further modifications are required.

Best Regards，

Dr. Xuhua Yu
